# Supplementary material for: Spatial close-kin mark-recapture methods to estimate dispersal parameters and barrier strength for mosquitoes
Source: PLoS Comput Biol. 2025 Nov 14;21(11):e1013713. doi: 10.1371/journal.pcbi.1013713 (PMC12638032; doi:10.1371/journal.pcbi.1013713)
Supplement: Attachment — Submitted filename: PCOMPBIOL-D-25-00982_Comments.pdf [file pcbi.1013713.s002.pdf]

Review of PCOMPBIOL-D-25-00982 "Spatial close-kin mark-recapture methods to estimate dispersal parameters and barrier strength for mosquitoes"

<u>Major comments:</u>

I found this paper to be a thorough and enjoyable read, owing in no small part to the clarity of the writing. I am not a mosquito biologist, but I am familiar with close-kin mark-recapture and found the methods to be well-described and appropriate. From a CKMR perspective the described work represents a useful contribution to the literature. I am less able to critique this work's place in the larger body of literature concerning mosquito dispersal and methods for its estimation, but engagement with other contemporary works (e.g., Jasper et al. 2019 and Filipovic et al. 2020) seemed appropriate. I had a few minor comments (detailed below) but detected no fundamental flaws or issues with either the analyses or presentation of results

<u>Minor comments:</u>

Author summary: The first line is a key statement and I believe it can be improved. Strictly speaking, CKMR does not deal with marked individuals but *identified or inferred* ones. Suggest something along the lines of "in which the frequency of *identified or inferred parents* in a sample is used to infer demographic parameters such as census population size and mean dispersal distance

Line 24: misplaced comma

Line 48: Re-using paragraph opening from previous paragraph is rather jarring. I see that a different point is being made, but some rewording of either topic sentence (I suggest the preceding one) will improve readability.

Line 74: For the population to be at equilibrium, wouldn't the emergence rate have to equal the total mortality rate? I.e., the combination of Ua, Ue, Up, etc? Or am I misinterpreting something here.

Line 289: To avoid double-counting and self-counting. The latter is implied by the equation and preceding sentence, but may still be useful to mention.

Line 365: Wrong tense?

Figures 4 & 5: Is color necessary here? If so, consider switching to a colorblind-friendly scheme. Panels A and E in Fig. 4 are especially problematic

Line 519: Missing commas around "however"
