# Supplementary material for: Spatial close-kin mark-recapture methods to estimate dispersal parameters and barrier strength for mosquitoes
Source: PLoS Comput Biol. 2025 Nov 14;21(11):e1013713. doi: 10.1371/journal.pcbi.1013713 (PMC12638032; doi:10.1371/journal.pcbi.1013713)
Supplement: Attachment — Submitted filename: review_pcompbio.pdf [file pcbi.1013713.s003.pdf]

Main comment:

This study uses close-kin mark-recapture (CKMR) methods to estimate key dispersal parameters for mosquitoes, and employs simulations to identify optimal sampling schemes. This is a brilliantly written and well executed paper that falls into line with recent publications highlighting the versality and potential of CKMR to study mosquito dispersal (a topic with significant implications for understanding vector-borne diseases). I commend the authors for submitting a highly polished version in the first round of review (which is unfortunately rarely the case).

My background is more closely aligned with CKMR than mosquito biology, and so my main feedback focuses on enhancing the description of mosquito biology and its integration with CKMR. I provide detailed comments below line by line, in the order of the manuscript (or, sometimes in the order of my brain).

I hope these help. It was a real pleasure to read this study, well done!

Line-by-line comments:

+ L.73-74: It took me a second to understand what this sentence means. Essentially, you assume that each female mates only once, with a single male, and has multiple subsequent egg laying events (at different places and times)? This seems like a rather big assumption, with major importance for paternal kinship probabilities (and it would also essentially mean that half-siblings do not exist). I am not a mosquito biologist, but how realistic is this assumption, and is it transferable to many mosquito species of interest? Do you expect the probability of a female having multiple reproductive events that low because of low survival probabilities? A few sentences to justify this choice would help the reader (particularly those not familiar with mosquito life history).

+ L.123-125: This is another mosquito-naïve comment, but up until this point, I somehow imagined that the "dispersal" being studied here was natal dispersal (i.e., dispersal of an individual from place of birth to place of capture). It might be worth mentioning (even if it seems obvious to you) that you are talking about adult dispersal, which consists of multiple events between habitat patches.

+ L.123-125: Another point of confusion for me was the use of t_1 and t_2 in reverse order. Since sampling is lethal, then t_1 (sampling of adult) has to come *after* t_2 (egg laying), correct? I suppose you want to use *1* for adult and *2* for offspring (since you are already using *i* and *j* for dispersal), but this creates quite a bit of confusion (my first instinct was to think that t_2 came after t_1).

+ Fig 2: Excellent figure to help understand the kinship probability. Well done!

+ Equation 7: At first read, I was missing the link between m_{ij} and ψ_{MOL}. On second read, I realised that you mentioned that the daily movement matrix M was a function of m_{ij} much earlier in the text (lines 923-93). I think a quick reminder here, when describing the role of ψ_{MOL} in the kinship probabilities, would be helpful to the reader (no need to redefine m_{ij} entirely).

+ L. 315-327: Apologies if I missed it, but it is not clear how you account for the barrier strength in the dispersal kernel (and, by extension, the formula of ψ_{MOL})?

+ L. 315-327: Another question related to the effect of the barrier. Can this framework be used to identify barriers, or do you need to have barrier information a priori in the calculation of m_{ij} (i.e., know which pairs *ij* are affected by δ, and which are not)?

+ L. 344: Biweekly as in twice a week or once every two weeks?

+ Figure 4: I think there is a good argument to simplify figure 4. I do not think plots A and B are very useful, since the results change once you incorporate the findings of C and reassess total sample size and life stage in D and E.

At first, I was left wondering why I was looking at two copies of the same figure (A-E and B-D) with slightly different results. I was also wondering what went wrong in your simulations/methods to find a constant 10-15% underestimation of $1/\lambda_d$ in all of your results. Only after reading about trap grid size and spacing (C) did I understand what the issue was, and that D and E showed the relative effects of life stage and sample size with "optimal" grid size. In other words, you were testing bias caused by one parameter whilst another parameter was itself creating additional bias.

Could you remove A and B, start with C with the default sampling scheme (since sample size does not seem to have a big effect), and then assess life stage and sample size with optimal grid size and spacing (as you did for D and E)? I think this would simplify the results significantly (or you could also include A and B in supplementary information, if you do not want to remove it).

+ Figure 4: As you mentioned, "the 5-by-5 grid of trap nodes with each trap node separated by two population nodes represents the largest number of traps that can be accommodated on the simulated landscape for this degree of separation." Have you checked what would happen if the spacing between traps was too large (e.g., in a scenario where the dispersal of the species in this area is gravely misjudged, and the spacing between traps is 1-2x mean lifetime dispersal)? I presume that you would find no MOP, but you could still find full-siblings. How would this affect your results?

+ L.443-445: Even more questions about the barrier! Would that 3-5% bias vary with barrier configuration? Thinking for instance if a barrier is in the middle of the population, or nearer to the edge (which would influence the average distance to the barrier from the different locations). I think this 3-5% bias is only reflective of the parameters in your simulations, and it should be made clear that it could be smaller or larger in other configurations (I imagine).

+ L.450-451: More out of curiosity than a suggestion: did you consider an alternative formulation where you have residents vs dispersers? Are those two common "lifetime strategies", or are *Ae. aegypti* that remain in the same location more flexible and adapt to surrounding resources at time t?

+ L.465-469: So, the bias in estimation of p_0 found for low values of p_0 is inherent to the exponential dispersal kernel used in the model. How would you address this issue in a real-life application, if you suspected to be in this scenario? Can you give a suggestion (e.g., use a different dispersal kernel)?

Actually, this point is valid for other biases found in your study (e.g., underestimation of $1/\lambda_d$ for high values of barrier strength). You mention the biases, the potential reasons for them, but you do not give recommendations/suggestions to either identify or correct them.

+ L.493-498: Is there a way to suggest a "golden" sample size for different species, population size, and spatial setting of the landscape. For example, saying that "traps in this configuration were separated by approximately the simulated lifetime dispersal distance" is great because the same threshold likely applies to different species, disregarding of their actual dispersal kernel. Could you do the same for sample size (n% of total population size / number of grid), or would you rather recommend each study to explore their best sampling strategies through ad-hoc simulations?

+ L.545-547: Let's imagine a situation where none of the demographic parameters are known. How do you think the estimation of dispersal parameters would fair? Would precision be mostly impacted,

or can you see scenarios where a potential bias might occur if another demographic parameter is poorly estimated?

+ L.549: I would call these assumptions more than limitations. These assumptions are typically what one would expect from a simulation study. Someone wishing to apply these methods in the field should be aware that real life is always more complex.

+ L.573: Again, as a non-mosquito-expert, it would be great to read more about why it is important to estimate dispersal of mosquitoes, and why CKMR can be a good addition to the already-existing methods.

+ L.584-586: What if we do not know the mean lifetime dispersal distance of the species in our study area? Or is that information usually available for comparable species/systems?
