# Supplementary material for: Spatial close-kin mark-recapture methods to estimate dispersal parameters and barrier strength for mosquitoes
Source: PLoS Comput Biol. 2025 Nov 14;21(11):e1013713. doi: 10.1371/journal.pcbi.1013713 (PMC12638032; doi:10.1371/journal.pcbi.1013713)
Supplement: Attachment — Submitted filename: MarshallEtAl_2025_R1_ResponseToReviewers.pdf [file pcbi.1013713.s005.pdf]

Berkeley Public Health

UNIVERSITY OF CALIFORNIA, BERKELEY

DIVISIONS OF BIOSTATISTICS & EPIDEMIOLOGY
SCHOOL OF PUBLIC HEALTH
2121 BERKELEY WAY #5302
BERKELEY, CA 94720

October 29, 2025

Dear Drs. Tyler Cassidy and Tobias Bollenbach,

Please find enclosed our revised manuscript entitled, "Spatial close-kin mark-recapture methods to estimate dispersal parameters and barrier strength for mosquitoes" for consideration as a research article in PLoS Computational Biology. We greatly appreciate the round of reviewers' comments and believe we now have a significantly refined manuscript thanks to their thoughtful comments. We have constructively addressed each point below, with reviewer comments in black text, our response in blue text, and quotations from the revised manuscript in blue italics text and quotation marks. We thank you and the three reviewers for your time and for improving the quality of our manuscript. We look forward to hearing your response regarding the revised manuscript.

Sincerely,

John Marshall, PhD, on behalf of the authors

Professor
School of Public Health
University of California, Berkeley
Email: john.marshall@berkeley.edu
Website: https://www.marshalllab.com/

Reviewer comments:
****

Editors:

Comments:

This is an interesting and well-presented manuscript. In general, the reviewers appreciated the approach and using CKMR to understand mosquito dispersal. However, both Reviewer 1 and 3 both noted figure 4 as being difficult to interpret (and not colourblind friendly). Further, Reviewer 3's comments on identifiability of model parameters and computational cost are important to address, given the presentation of the pseudo-likelihood in Section 2.3, and Reviewer 1's comments regarding Lines 493-498 and Lines 545-547. Finally, while many of the comments are minor, all three reviewers have made suggestions regarding additional analysis/situations. In the revision, please consider performing the additional analyses that may highlight the limitations or feasibility of the CKMR approach to be used in practice.

We thank the editors and reviewers for their thorough reading of the manuscript, and their constructive comments to improve the analyses and text. We have addressed all reviewer comments to the best of our ability, and have conducted several additional analyses, per the reviewers' recommendations, that we agree enhance understanding of the feasibility and limitations of the CKMR approach. We have also addressed comments regarding identifiability of model parameters and computational cost, and have made the suggested changes to Figure 4 to address concerns regarding ease of interpretation and colorblind-friendliness.

Reviewer #1:

Major comments:

I found this paper to be a thorough and enjoyable read, owing in no small part to the clarity of the writing. I am not a mosquito biologist, but I am familiar with close-kin mark-recapture and found the methods to be well-described and appropriate. From a CKMR perspective the described work represents a useful contribution to the literature. I am less able to critique this work's place in the larger body of literature concerning mosquito dispersal and methods for its estimation, but engagement with other contemporary works (e.g., Jasper et al. 2019 and Filipovic et al. 2020) seemed appropriate. I had a few minor comments (detailed below) but detected no fundamental flaws or issues with either the analyses or presentation of results.

Thank you for your feedback and close reading of the manuscript. We are happy to hear that you found the paper to be well-written and an enjoyable read.

Minor comments:

Author summary: The first line is a key statement and I believe it can be improved. Strictly speaking, CKMR does not deal with marked individuals but identified or inferred ones. Suggest something along the lines of "in which the frequency of identified or inferred parents in a sample is used to infer demographic parameters such as census population size and mean dispersal distance.

Thanks for catching this. We have edited this sentence to read: *"Close-kin mark-recapture (CKMR) is a genetic analogue of mark-release-recapture (MRR) in which the frequency of genetically-inferred familial relationships in a sample is used to infer demographic parameters…"*

Line 24: misplaced comma

We have edited this sentence according to the Reviewer's suggestion: *"...inferred dispersal patterns may be modified by the process of marking and capturing; for mosquitoes specifically, releasing females may increase the risk of local disease transmission."*

Line 48: Re-using paragraph opening from previous paragraph is rather jarring. I see that a different point is being made, but some rewording of either topic sentence (I suggest the preceding one) will improve readability.

We agree and have edited the opening of the previous paragraph to avoid the issue that the Reviewer points out. The modified opening now reads *"Close-kin mark-recapture (CKMR) is a promising new approach with the potential to complement these methods and deepen understanding of mosquito dispersal."*

Line 74: For the population to be at equilibrium, wouldn't the emergence rate have to equal the total mortality rate? I.e., the combination of Ua, Ue, Up, etc? Or am I misinterpreting something here.

This is correct as currently stated. For the population to be at equilibrium, the rate of adult female emergence (or the rate of adult emergence, generally) must equal the adult female mortality rate (or the adult mortality rate, generally). The other parameters of the lumped age class model, depicted in Figure 1A, facilitate this equilibrium - i.e., the number of eggs laid per day, and the duration and mortality rate of each juvenile life stage, result in an adult (female) emergence rate that matches the adult (female) mortality rate.

Line 289: To avoid double-counting and self-counting. The latter is implied by the equation and preceding sentence, but may still be useful to mention.

Thank you, we have now referred to self-counting as well as double-counting.

Line 365: Wrong tense?

Thank you for spotting this, we have corrected it.

Figures 4 & 5: Is color necessary here? If so, consider switching to a colorblind-friendly scheme. Panels A and E in Fig. 4 are especially problematic

Thank you for this suggestion. Figures 4-6 in the manuscript and Figures S3 and S4 in the S1 Text have been switched to a colorblind-friendly scheme.

Line 519: Missing commas around "however"

Thank you for spotting this, we have corrected it.

Reviewer #2:

Main comment:

This study uses close-kin mark-recapture (CKMR) methods to estimate key dispersal parameters for mosquitoes, and employs simulations to identify optimal sampling schemes. This is a brilliantly written and well executed paper that falls into line with recent publications highlighting the versatility and potential of CKMR to study mosquito dispersal (a topic with significant implications for understanding vector-borne diseases). I commend the authors for submitting a highly polished version in the first round of review (which is unfortunately rarely the case).

Thank you for your close reading of the paper. We are happy to hear that it was well received.

My background is more closely aligned with CKMR than mosquito biology, and so my main feedback focuses on enhancing the description of mosquito biology and its integration with CKMR. I provide detailed comments below line by line, in the order of the manuscript (or, sometimes in the order of my brain). I hope these help. It was a real pleasure to read this study, well done!

Thank you for your constructive comments. They are very helpful!

Line-by-line comments:

+ L.73-74: It took me a second to understand what this sentence means. Essentially, you assume that each female mates only once, with a single male, and has multiple subsequent egg laying events (at different places and times)? This seems like a rather big assumption, with major importance for paternal kinship probabilities (and it would also essentially mean that half-siblings do not exist). I am not a mosquito biologist, but how realistic is this assumption, and is it transferable to many mosquito species of interest? Do you expect the probability of a female having multiple reproductive events that low because of low survival probabilities? A few sentences to justify this choice would help the reader (particularly those not familiar with mosquito life history).

Thanks for pointing out the importance of clarifying mosquito mating behavior. It is common, when modeling mosquito population dynamics, to ignore female re-mating, because this happens at such low frequencies. We have added a clause and two references to the relevant sentence in the Methods section to clarify this:

"Given the rareness of female re-mating for important mosquito vector species (<1% for An. gambiae (Tripet et al., 2003) and <1-5% for Ae. aegypti (Richardson et al., 2015)), we assume that females mate once upon emergence, and retain the genetic material from that mating event for the remainder of their lives."

We have avoided going into too much detail in this section of the manuscript, so as not to distract from other details of the mosquito life history model; but the Reviewer is correct that this influences kinship probabilities - it means that maternal half-siblings (same father, different mothers) are not possible; but that paternal half-siblings (same mother, different fathers) are. We have added mention of this in the opening paragraph of the Results section:

"Half-siblings and higher-order kinship pairs could be included for individual data analyses; however, computational burden prevented us from including these for exploratory analyses (note that only paternal half-siblings are possible, since we assume that females mate only once)."

+ L.123-125: This is another mosquito-naïve comment, but up until this point, I somehow imagined that the "dispersal" being studied here was natal dispersal (i.e., dispersal of an individual from place of birth to place of capture). It might be worth mentioning (even if it seems obvious to you) that you are talking about adult dispersal, which consists of multiple events between habitat patches.

We appreciate these suggestions that ensure the paper is understandable to non-mosquito biologists. We have added the following sentence to section 2.2 introducing the kinship probabilities:

"Furthermore, mosquito dispersal is restricted to the adult life stage, and hence displacement between close-kin pairs represents an accumulation of adult movements between events such as emergence, egg-laying and capture, etc."

+ L.123-125: Another point of confusion for me was the use of t1 and t2 in reverse order. Since sampling is lethal, then t1 (sampling of adult) has to come *after* t2 (egg laying), correct? I suppose you want to use 1 for adult and 2 for offspring (since you are already using i and j for dispersal), but this creates quite a bit of confusion (my first instinct was to think that t2 came after t1).

We have chosen to retain the indexing of individuals as "1" and "2," and their respective capture times as t_1 and t_2, for consistency with our prior analysis in Sharma *et al.* (2022). Here, parent-offspring kinship probabilities are described as "the probability that an individual sampled on day t_2 is the offspring of an adult sampled on day t_1." As depicted in Figure 3 of Sharma *et al.* (2022), in fact, t_1 may come either before or after t_2. For mother-larval offspring pairs, it is more likely that t_1 comes after t_2; but for all other parent-offspring pairs depicted in Figure 3 of Sharma *et al.* (father-larval offspring, father-adult offspring, and mother-adult offspring) it is more likely that t_2 comes after t_1.

+ Fig 2: Excellent figure to help understand the kinship probability. Well done!

Thank you, we are glad that this is helpful in aiding understanding.

+ Equation 7: At first read, I was missing the link between mij and ψMOL. On second read, I realised that you mentioned that the daily movement matrix M was a function of mij much earlier in the text (lines 92-93). I think a quick reminder here, when describing the role of ψMOL in the kinship probabilities, would be helpful to the reader (no need to redefine mij entirely).

The following sentence has been added to this section to remedy this:

"Recall that the entries of the movement matrix, M, are m_{ij}, as defined in Equations 1 and 2 for the exponential and zero-inflated exponential dispersal kernel, respectively."

+ L. 315-327: Apologies if I missed it, but it is not clear how you account for the barrier strength in the dispersal kernel (and, by extension, the formula of ψMOL)?

It's true that the incorporation of barrier strength within the dispersal kernel was not properly defined in the original submission. We have now expanded upon this in section 2.1 of the Methods section:

"In some simulations and analyses, a barrier to movement is included (Figure 1D), which could represent a road, freeway or open park space. In this case, prior to normalization, the movement rate between populations i and j is reduced by a factor equal to the barrier strength, δ, for populations on opposite sides of the barrier, and is unchanged for populations on the same side of the barrier. Movement rates are then normalized again so that they sum to 1 for each origin, i."

+ L. 315-327: Another question related to the effect of the barrier. Can this framework be used to identify barriers, or do you need to have barrier information a priori in the calculation of mij (i.e., know which pairs ij are affected by δ, and which are not)?

We explore this question in a follow-up manuscript, currently being drafted, where we apply spatial CKMR methods to *Ae. aegypti* mosquitoes collected in Singapore on either side of a six-lane freeway. In brief, for field-collected mosquitoes, it is likely there would be a small number of known potential barriers in the landscape. For each potential barrier, parameters could be estimated (e.g., barrier strength), and the likelihood of the kinship data given the model could be calculated. By comparing likelihoods across models incorporating different barrier set-ups, a preferred model could be selected according to a model selection criterion such as the Akaike Information Criterion. We will save this elaboration for the follow-up paper.

+ L. 344: Biweekly as in twice a week or once every two weeks?

True, the word "biweekly" is ambiguous. We have clarified the definition used in this paper following its first usage, in the opening paragraph of the Results section:

"...sampling frequency was biweekly (i.e., twice per week, as is often the case for mosquito surveillance programs (Ndiaye et al., 2018))..."

+ Figure 4: I think there is a good argument to simplify figure 4. I do not think plots A and B are very useful, since the results change once you incorporate the findings of C and reassess total sample size and life stage in D and E. At first, I was left wondering why I was looking at two copies of the same figure (A-E and B-D) with slightly different results. I was also wondering what went wrong in your simulations/methods to find a constant 10-15% underestimation of 1/λd in all of your results. Only after reading about trap grid size and spacing (C) did I understand what the issue was, and that D and E showed the relative effects of life stage and sample size with "optimal" grid size. In other words, you were testing bias caused by one parameter whilst another parameter was itself creating additional bias. Could you remove A and B, start with C with the default sampling scheme (since sample size does not seem to have a big effect), and then assess life stage and sample size with optimal grid size and spacing (as you did for D and E)? I think this would simplify the results significantly (or you could also include A and B in supplementary information, if you do not want to remove it).

We thank the Reviewer for this suggestion. We have removed panels A and B from Figure 4, and moved them to the S1 Text, which now includes a supplemental results section (§3). Supplemental optimal sampling scheme results are now included in §3.1 and Figure S3 in the S1 Text.

+ Figure 4: As you mentioned, "the 5-by-5 grid of trap nodes with each trap node separated by two population nodes represents the largest number of traps that can be accommodated on the simulated landscape for this degree of separation." Have you checked what would happen if the spacing between traps was too large (e.g., in a scenario where the dispersal of the species in this area is gravely misjudged, and the spacing between traps is 1-2x mean lifetime dispersal)? I presume that you would find no MOP, but you could still find full-siblings. How would this affect your results?

We thank the Reviewer for another great suggestion. To address this, we have added a new panel D to Figure 4, in which we explored trap spacing on a larger 37-by-37 grid so that the trap nodes in a 5-by-5 grid can be separated by up to seven household nodes each, with two household nodes reserved as a buffer at each boundary. The following paragraph describing these results has been added to §3.1 of the manuscript:

"Considering the optimal sampling scheme corresponds to the case where a 5-by-5 grid of traps is spread out as much as possible through a 19-by-19 grid of households (Figure 1D), we conducted a supplemental analysis in which trap nodes were separated by 0-7 population nodes, to see how far apart traps may be placed while still obtaining reasonable estimates of mean daily dispersal distance. To achieve this, we carried out 100 simulation-and-analysis replicates for each scenario on a larger 37-by-37 household grid, in order to accommodate the maximum trap separation and provide a two-house buffer at each landscape boundary. Results, depicted in Figure 4D, suggest that estimates of $1/\lambda_d$ are reasonably accurate (i.e., the IQR encompasses the true value) for trap node separations of 2-7 household nodes, although the accuracy (as measured by the distance between the median of 100 simulations and the true value) begins to decline for trap node separations of 6-7 household nodes. This suggests that accurate estimation of $1/\lambda_d$ may be obtained for trap nodes placed a distance of 1-2.5 times the mean lifetime

dispersal distance apart. Conveniently, this means that the dispersal behavior of the species need only be approximately known when designing a sampling scheme."

Interestingly, these results imply that related individuals are still found at distances ~2.5 times the mean lifetime dispersal distance apart and beyond due to $1/\lambda_d$ representing a mean dispersal distance, and some individuals dispersing further than this. Indeed, further inspection of the results does reveal fewer related pairs being found at larger separation distances, which correlates with the slight reduction in precision of $1/\lambda_d$ estimates for trap nodes separated by 6-7 population nodes.

+ L.443-445: Even more questions about the barrier! Would that 3-5% bias vary with barrier configuration? Thinking for instance if a barrier is in the middle of the population, or nearer to the edge (which would influence the average distance to the barrier from the different locations). I think this 3-5% bias is only reflective of the parameters in your simulations, and it should be made clear that it could be smaller or larger in other configurations (I imagine).

Inspired by this comment, we conducted an additional analysis to explore the impact of barrier location on the estimation of barrier strength. This has now been included in §3.2 of the S1 Text, which reads:

"Given the optimal sampling scheme described in section §3.1 of the manuscript, we employ the flexibility of the formal CKMR approach to estimate parameters describing the strength of a barrier to movement, δ, for two barrier locations: i) a barrier near the "center" of the landscape (at the midpoint between the third and fourth row of traps, as depicted in Figure 1D in the manuscript), and ii) a barrier near the edge of the landscape (at the midpoint between the fourth and fifth row of traps). We explore δ values of 0.75 and 0.9 for each barrier location and adopt the optimal sampling scheme determined in §3.1 of the manuscript - a total sample of 2,500 adult females sampled from a 5-by-5 grid of trap nodes with two population nodes separating each trap node. Results in Figure S4 suggest that estimates of δ are equally accurate (as measured by the distance between the median of 100 simulations and the true value) for values of δ ≥ 0.75 regardless of barrier location, and that barrier strength estimates are more precise (as measured by IQR) for δ values of 0.9 as compared to 0.75."

Results are depicted in Figure S4 in the S1 Text, and the following reference has been made to these results in §3.2 of the manuscript:

"Fortunately, barrier location does not present an obstacle to parameter inference, as we demonstrate in S1 Text §3.2."

+ L.450-451: More out of curiosity than a suggestion: did you consider an alternative formulation where you have residents vs dispersers? Are those two common "lifetime strategies", or are Ae. aegypti that remain in the same location more flexible and adapt to surrounding resources at time t?

Given the structure of our model, each mosquito was modeled as following the same "rules," so we didn't separate "residents" vs. "dispersers," explicitly. However, the zero-inflated exponential kernel does have a very high daily staying probability - up to 90% - and given the grid-based nature of our landscape, even the exponential kernel has an effective daily staying probability of ~30%. Combined with a daily adult mosquito mortality rate of 9%, this results in a significant number of mosquitoes effectively acting as residents, even if they had the "option" to disperse each day.

+ L.465-469: So, the bias in estimation of p0 found for low values of p0 is inherent to the exponential dispersal kernel used in the model. How would you address this issue in a real-life application, if you suspected to be in this scenario? Can you give a suggestion (e.g., use a different dispersal kernel)?

We have added the following text to the end of the second paragraph of section 3.2:

"For field data where a value of p_0 ≤ 0.5 is inferred, we advise also fitting an exponential dispersal kernel and performing model selection."

Essentially, an exponential dispersal kernel is not very different from a zero-inflated exponential dispersal kernel for values of p_0 ≤ 0.5, and hence the exponential kernel may indeed provide a more parsimonious fit.

Actually, this point is valid for other biases found in your study (e.g., underestimation of 1/λd for high values of barrier strength). You mention the biases, the potential reasons for them, but you do not give recommendations/suggestions to either identify or correct them.

We don't have a recommendation regarding estimation of $1/\lambda_d$ for high values of barrier strength; but we do have a recommendation regarding estimation of δ for low estimates of barrier strength, which we have now added to the first paragraph of section 3.2::

"For field data where a value of $\delta \leq 0.25$ is inferred, we advise also fitting a model without a barrier present and performing model selection."

A model with a weak barrier may not be very different from a model with no barrier, and a model with no barrier may provide a more parsimonious fit.

+ L.493-498: Is there a way to suggest a "golden" sample size for different species, population size, and spatial setting of the landscape. For example, saying that "traps in this configuration were separated by approximately the simulated lifetime dispersal distance" is great because the same threshold likely applies to different species, disregarding of their actual dispersal kernel. Could you do the same for sample size (n% of total population size / number of grid), or would you rather recommend each study to explore their best sampling strategies through ad-hoc simulations?

The following sentence has been added to the second paragraph of the Discussion:

"Precise sample size requirements should ideally be inferred by repeating simulation and analysis replicates for the species and landscape of interest."

Indeed, it is possible that there could be a "golden" sample size for different species and landscapes. We thought about this; but were not able to derive something general at this time - this could be an area of future research. Notably, Bravington *et al.* (2016) describe a sample size on the order of the square root of N to estimate census population size for a panmictic population; however, life history and spatial structure introduce heterogeneities that complicate this calculation, and the nature of the dispersal kernel may also play a role.

+ L.545-547: Let's imagine a situation where none of the demographic parameters are known. How do you think the estimation of dispersal parameters would fair? Would precision be mostly impacted, or can you see scenarios where a potential bias might occur if another demographic parameter is poorly estimated?

It is feasible that precision and/or bias of dispersal parameters could be impacted by poorly characterized life history or landscape parameters, and sensitivity analyses would be the best approach to determine the magnitude of these effects. To address this comment, we have modified the end of the paragraph referred to to read:

"A sensitivity analysis on life history and other parameters at the simulation stage could determine impacts on the precision and bias of dispersal parameters in the event that other parameters are poorly characterized. That said; the formal CKMR approach can also be used to estimate demographic parameters unrelated to dispersal, such as census population size, adult and larval daily mortality rates, and larval life stage duration, as shown in Sharma et al. (2022), which could themselves inform spatial CKMR analyses to estimate dispersal parameters."

+ L.549: I would call these assumptions more than limitations. These assumptions are typically what one would expect from a simulation study. Someone wishing to apply these methods in the field should be aware that real life is always more complex.

We agree that these are both assumptions and limitations. Stylistically, we prefer to describe them primarily as assumptions in the Methods section, and primarily as limitations in the Discussion section - i.e., the fact that we relied upon these assumptions to conduct our study represents a set of limitations that we discuss here.

+ L.573: Again, as a non-mosquito-expert, it would be great to read more about why it is important to estimate dispersal of mosquitoes, and why CKMR can be a good addition to the already-existing methods.

This is already covered reasonably extensively in the Introduction. The first paragraph details why it is important to characterize mosquito dispersal, the second paragraph describes already-existing methods, and the third and fourth paragraphs detail why CKMR might be a useful complementary approach to currently-available methods.

+ L.584-586: What if we do not know the mean lifetime dispersal distance of the species in our study area? Or is that information usually available for comparable species/systems?

Per the response to an earlier comment, our subsequent analysis (now included in Figure 4, panel D) illustrates that traps should be separated by a distance of at least the mean lifetime dispersal distance; but may also be separated by a distance up to 2.5 times the mean lifetime dispersal distance. This relatively wide window of suitable trap separation distances means that we don't need to know the mean lifetime dispersal distance of the species of interest in our study area precisely. Additionally, estimates of mosquito dispersal parameters are widely available from studies such as mark-release-recapture (MRR), albeit with the inherent biases associated with marking and recapturing listed in the Introduction. We therefore edited the relevant sentence in the Conclusions section accordingly:

"In our in silico case study, we found that traps distributed in a grid layout and separated by a distance of approximately 1-2.5 times the mean lifetime dispersal distance of this species were optimal."

Edits have also been made throughout the manuscript to this effect, including in the Abstract, Results and Discussion sections. For example, in the Results section, we now state:

"This suggests that accurate estimation of $1/\lambda_d$ may be obtained for trap nodes placed a distance of 1-2.5 times the mean lifetime dispersal distance apart. Conveniently, this implies that the dispersal behavior of the species need only be approximately known when designing a sampling scheme."

Reviewer #3:

Overall comments:

This manuscript explores use CKMR methods to characterise mosquito dispersal patterns by simulation. The manuscript is well written and flows well. I have a few major comments and minor comments that maybe helpful to strengthen the manuscript.

Thank you very much for your close reading of the manuscript and constructive comments for its improvement.

Major comments.

1. The authors assume perfect kinship inference throughout. Have the authors done a sensitivity analysis to make sure this is reasonable. I see they have presented some references related to this, but conducting a sensitivity analysis would certainly strengthen the analysis.

We thank the Reviewer for this suggestion. In response, we have added section 3.1.1 to the manuscript, entitled "False negative and false positive kinship inference." The text of this section is included below:

"We explored the role that false negative and false positive kinship inference could have on estimation of $1/\lambda_d$. To investigate this, we used the optimal sampling scheme determined above for a 19-by-19 household grid, and performed 100 simulation replicates for scenarios in which: i) 0-20% of mother-offspring and full-sibling pairs were decoupled at random (i.e., introducing false negatives), and ii) 0-20% of individuals without sampled mothers or full-siblings were assigned them at random (i.e., introducing false positives). Results, depicted in Figure 5, show that introduction of false negatives, even at proportions as high as 20%, has little impact on the estimated value of $1/\lambda_d$ (Figure 5A). Introduction of

false positives, however, causes an upwards bias in estimates of $1/\lambda_d$ by a degree proportional to the number of false positives in the dataset (Figure 5B). This is likely because false positives tend to be separated by larger distances than true positives. False negatives, on the other hand, convey little information about dispersal patterns. Based on these results, we recommend aiming for a false positive kinship inference rate of less than 5%, and erring towards inference of false negatives rather than false positives where trade-offs exist."

Results of this analysis are depicted in a new Figure 5, with false negatives included in panel A, and false positives included in panel B. The following text in the Discussion section has also been updated to reflect these results:

"Fortunately, through introducing kinship assignment errors at the simulation phase, we have shown that inference of mean dispersal distance is robust to type II (false negative) errors, and can accommodate type I (false positive) errors of up to 5%. This suggests that, where trade-offs in kinship assignment are possible, it is best to err towards false negatives rather than false positives, in agreement with CKMR studies in fish species (Bravington et al., 2016)."

2. The manuscript has not discussed potential identifiability issues if any. Would be great if the authors can add a small discussion/analysis on this.

We have added the following text to section 2.3.4 on parameter inference to address this comment:

"Parameter identifiability was not an apparent issue in our analyses; however, Bravington et al. (2016) note that the Fisher information matrix can be used to study parameter identifiability in CKMR analyses, and that the per-pair Fisher information can provide a valuable tool for sampling scheme design."

3. CKMR method seem to be computationally intense. If so, how applicable is this in general and for larger populations or higher-order kinship?

The following sentence has been edited in the first paragraph of the Results section to describe barriers to inclusion of half-siblings and higher-order kinship pairs in this formulation of CKMR:

"Half-siblings and higher-order kinship pairs could be included for individual data analyses; however, computational burden prevented us from including these for exploratory analyses (note that only paternal half-siblings are possible, since we assume that females mate only once)."

That is to say that, since we were conducting thousands of simulation-and-analysis replicates as part of this study, we were required to limit the computational burden of each replicate; however, were a field dataset to be analyzed, higher-order kinship pairs could be included because this would only be a single analysis. Regarding the scaling of computational intensity with population size, these methods scale in intensity with sample size. The following sentence has been added to section 2.3.2 of the Methods, where the pseudo-likelihood calculation is described for full-sibling pairs:

"Full-sibling pairs represent the majority of the computational burden of the pseudo-likelihood calculation, and from the above equation, this can be seen to scale approximately linearly with total sample size."

4. The simulations are based on a regular grid landscape, which may not be reflective of the real-world heterogeneity. Would be great if the authors can add a discussion point on this.

The following text has been added to the second-to-last paragraph of the Discussion, where limitations of the study are discussed:

"The grid landscape, with each population having the same equilibrium population size, also represents an overly simplified scenario that does not capture the heterogeneity of real landscapes. That said; this is an appropriate starting point to verify the utility of the method, beyond which further research may be conducted."

5. The comparisons with alternative methods done in the manuscript are mostly qualitative. Perhaps the authors could add a quantitative comparison (bias, variance, etc) using simulated data to better understand the trade-offs.

We actually did conduct a quantitative comparison of the three mosquito close-kin-based methods in an earlier draft of the manuscript; but then decided to remove it. The main issue was that the three methods required very different sampling schemes, and the performance of the methods depended on the framework used to assess them - e.g., ours performed best on a grid landscape, while the approach of Jasper *et al.* (2019) performed best in continuous space. The approaches of Jasper *et al.* (2019) and Filipović *et al.* (2020) also required larger sampling areas and hence larger landscapes to be simulated. A fair quantitative comparison of the methods would therefore require a substantial set of analyses to first optimize the sampling scheme for each method separately, and then to compare metrics (bias, variance, etc.) under the different optimal sampling scenarios. We considered this to be beyond the scope of this paper, and hence opted to include a qualitative comparison of the three methods instead. We agree with the Reviewer that a quantitative comparison of the methods would be a worthy topic for a separate manuscript.

Minor comments:

1. Figures 4 and 5 are not clear. Consider using colour-blind friendly colours with thicker lines for (or fill) the violin plots.

We have made the suggested changes to improve the clarity of Figures 4-6 in the manuscript, as well as to Figures S3 and S4 in the S1 Text.

2. The manuscript is well-cited, but perhaps authors can add more recent references on applications of CKMR if available.

Thank you for this suggestion. In response, we scanned the literature for recent applications of CKMR and other close-kin-based methods and added two recent papers - Ontiveros *et al.* (2025) and Patterson *et al.* (2025) - in addition to the recent Sévêque *et al.* (2025) paper, which we had already included. The Ontiveros *et al.* (2025) paper is a nice addition, as although not a formal CKMR paper, it applies to the mosquito disease vector, *Aedes albopictus*.

References:
****

- Bravington MV, Skaug HJ, Anderson EC. Close-kin mark-recapture. Statistical Science. 2016; 31:259-274
- Filipović I, Hapuarachchi HC, Tien WP, Razak MA, Lee C, Tan CH, Devine GJ, Rašić G. Using spatial genetics to quantify mosquito dispersal for control programs. BMC Biology. 2020; 18:1-5.
- Jasper M, Schmidt TL, Ahmad NW, Sinkins SP, Hoffmann AA. A genomic approach to inferring kinship reveals limited intergenerational dispersal in the yellow fever mosquito. Molecular Ecology Resources. 2019; 19:1254-1264.
- Ndiaye EH, Diallo D, Fall G, Ba Y, Faye O, Dia I, et al. Arboviruses isolated from the Barkedji mosquito-based surveillance system, 2012-2013. BMC Infectious Diseases. 2018;18: 642. pmid:30541472.
- Ontiveros V, Lucati F, Caner J, Timor AA, Eritja R, Pilosof S et al. Fine-scale tiger mosquito population dynamics in urban and densely populated landscapes. Authorea. 2025; doi:10.22541/au.174006032.26675184/v1.
- Patterson G, Goodfellow CK, Ting N, Kern AD, Ralph PL. Simulation-based spatially explicit close-kin mark-recapture. bioRxiv. 2025;doi: https://doi.org/10.1101/2025.05.31.656892.
- Richardson JB, Jameson SB, Gloria-Soria A, Wesson DM, Powell J. Evidence of limited polyandry in a natural population of Aedes aegypti . Am J Trop Med Hyg. 2015;93: 189-193.

- Sévêque A, Lonsinger RC, Waits LP, Morin DJ. Spatial close-kin mark-recapture models applied to terrestrial species with continuous natal dispersal. Methods in Ecology and Evolution. 2025;doi: 10.1111/2041-210X.14490.
- Sharma Y, Bennett JB, Rašić G, Marshall JM. Close-kin mark-recapture methods to estimate demographic parameters of mosquitoes. PLoS Comput Biol. 2022;18: e1010755.
- Tripet F, Touré YT, Dolo G, Lanzaro GC. Frequency of multiple inseminations in field-collected Anopheles gambiae females revealed by DNA analysis of transferred sperm. Am J Trop Med Hyg. 2003;68: 1-5.
