# Supplementary material for: Spatial close-kin mark-recapture methods to estimate dispersal parameters and barrier strength for mosquitoes
Source: PLoS Comput Biol. 2025 Nov 14;21(11):e1013713. doi: 10.1371/journal.pcbi.1013713 (PMC12638032; doi:10.1371/journal.pcbi.1013713)
Supplement: Attachment — Submitted filename: comments.docx [file pcbi.1013713.s004.docx]

Manuscript: Spatial close-kin mark-recapture methods to estimate

dispersal parameters and barrier strength for mosquitoes

overall comments: this manuscript explores use CKMR methods to characterise mosquito dispersal patterns by simulation. The manuscript is well written and flows well. I have a few major comments and minor comments that maybe helpful to strengthen the manuscript.

Major comments.

1. The authors assume perfect kinship inference throughout. Have the authors done a sensitivity analysis to make sure this is reasonable. I see they have presented some references related to this, but conducting a sensitivity analysis would certainly strengthen the analysis.
2. The manuscript has not discussed potential identifiability issues if any. Would be great if the authors can add a small discussion/ analysis on this.
3. CKMR method seem to be computationally intense. If so, how applicable is this in general and for larger populations or higher-order kinship?
4. The simulations are based on a regular grid landscape, which may not be reflective of the real-world heterogeneity. Would be great if the authors can add a discussion point on this.
5. The comparisons with alternative methods done in the manuscript are mostly qualitative. Perhaps the authors could add a quantitative comparison (bias, variance, etc) using simulated data to better understand the trade-offs.

Minor comments:

1. Figures 4 and 5 are not clear. Consider using colour-blind friendly colours with thicker lines for (or fill) the violin plots.
2. The manuscript is well-cited, but perhaps authors can add more recent references on applications of CKMR if available.
